# Distribution of gastrointestinal neuroendocrine tumors in Europe: results from a retrospective cross-sectional study

**DOI:** 10.1007/s00432-022-04003-3

**Published:** 2022-04-27

**Authors:** Sven H. Loosen, Karel Kostev, Henning Jann, Fabian Tetzlaff, Frank Tacke, Sarah Krieg, Wolfram T. Knoefel, Georg Fluegen, Tom Luedde, Andreas Krieg, Christoph Roderburg

**Affiliations:** 1 Clinic for Gastroenterology, Hepatology and Infectious Diseases, Medical Faculty, University Hospital Düsseldorf, Heinrich Heine University Düsseldorf, Moorenstrasse 5, 40225 Düsseldorf, Germany; 2 IQVIA, Frankfurt, Germany; 3 Department of Hepatology and Gastroenterology, Charité-Universitätsmedizin Berlin, Campus Virchow-Klinikum (CVK) and Campus Charité Mitte (CCM), Augustenburger Platz 1, 13353 Berlin, Germany; 4 Department of Surgery (A), Medical Faculty of Heinrich Heine, University Hospital Duesseldorf, Heinrich-Heine-University Duesseldorf, Moorenstraße 5, 40225 Düsseldorf, Germany

**Keywords:** Neuroendocrine tumors, NEN, Gastrointestinal, Epidemiology, Europe, Outcome

## Abstract

**Background:**

Gastrointestinal (non-pancreatic) neuroendocrine tumors (GI-NETs) represent a rare but increasingly common tumor entity. Prognosis and biological behavior of these tumors is extremely heterogenous and largely dependent on the specific tumor site, stage and differentiation. However, systematic data on the epidemiology of GI-NET, especially in terms of geographic distributions are missing.

**Methods:**

We used the Oncology Dynamics database (IQVIA) to identify a total of 1354 patients with GI-NET from four European countries (Germany, France, Spain, UK) and compared them with regard to major patient and tumor related characteristics including patients’ age, sex, tumor stage, tumor grading and differentiation.

**Results:**

Out of the analyzed 1354 NET patients, 535 were found in the UK (39.5%), 289 in Germany (21.3%), 283 in Spain (20.9%) and 247 in France (18.2%). More patients were male than female (53.8% vs. 46.2%) with no significant differences between the analyzed countries. In contrast, the age distribution varied between the different countries, with the highest number of patients identified in the age groups of 61–70 years (31.0%) and 71–80 years (30.7%). The vast majority of patients showed a tumor origin in the small intestine, in German patients NET of the large intestine were slightly overrepresented and NET of the stomach underrepresented compared to all other countries. More than 80% of patients had stage IV disease at the time of diagnosis. Regarding tumor histology, most tumors showed a G2 tumor; interestingly, a G3 grading was found in 40.9% of patients in Germany (Ki-67 > 20%).

**Conclusion:**

The distribution of important patient- and tumor-specific characteristics of neuroendocrine tumors shows regional differences in four major European countries. These data may help to better understand the specific epidemiology of GI-NET in Europe.

## Introduction

Neuroendocrine tumors of the gastrointestinal tract (GI-NETs) are a rare, heterogeneous group of tumors that originate from the diffuse endocrine system of different organs. Histologically, NENs show a strong (sometimes variable) expression of neuroendocrine markers including synaptophysin or chromogranin. NEN can occur in any part of the body. Next to their site of origin, the clinical behavior of NEN is widely dependent on the tumor differentiation. After the terminology of subdividing and classifying NEN has been adapted several times in the last decades, according to the current WHO classification, they are stratified based on their histological differentiation and on the Ki-67 proliferative index into low/ moderate- (grade 1/2, G1/2), high-grade (grade 3, G3) neuroendocrine tumors (NET) and neuroendocrine carcinomas (NEC) (Detjen et al. 2021; Rindi et al. 2018). While the poorly differentiated NET G3 are defined by a Ki-67 proliferation index of > 20% and a poor prognosis, well-differentiated NET (G1, G2) display low proliferation index, retain expression of somatostatin receptors (SSTR) and mostly feature excellent survival rates, which in some cases are similar to those of benign tumors (Detjen et al. 2021). Among all NET, GI-NET are a common representative of this tumor entity, with metastatic disease accounting for 12–22% of cases (Taal and Visser 2004). In almost all cases, NEN are sporadic, but association with the multiple endocrine neoplasia type 1 syndrome and clustering within families is known (Taal and Visser 2004). A slightly increased risk of secondary cancers was suggested by some authors (Taal and Visser 2004). In recent years, the incidence of NEN, including GI-NEN, has increased markedly in all parts of the world, which can be explained at least in part by a significant improvement in diagnostic modalities (Korse et al. 2012). Numerous studies point to regional variations in the distribution of NEN, although the data are very limited. The analysis of gastrointestinal (non-pancreatic) NEN incidence and characteristics of diagnosed NEN in four major European countries offers a unique opportunity to learn more about the epidemiology of these tumors. Since all of these countries have highly developed health care systems and bias due to different access to appropriate diagnostics and thus bias in incidence and tumor characteristics can be limited.

## Methods

### Database

This retrospective cross-sectional study is based on the data from IQVIA’s Oncology Dynamics (OD) database (Chambers et al. 2020; Marchetti et al. 2017; Zhao et al. 2012). This source is a cross-sectional partially retrospective survey collecting anonymized patient cases from a representative panel of oncologists. OD collects fully anonymized patient-level data on drug-treated cancer cases in several countries worldwide. Data collection and reporting is conducted through a standardized online questionnaire where all items are mandatory. A reporting manual with precise instructions on completing the questionnaire is provided to each respondent. Specific instructions are displayed through a ‘pop-up’ system throughout the survey to provide clear definitions for the desired variables. Physicians are also asked to enter factual information from the patient medical record to avoid recall bias. Further tactics to ensure input accuracy include controlled code lists and multiple-choice questions, as well as interactive filters that limit non-applicable questions (e.g., items on cancer-specific biomarkers). Responses are immediately validated against previous answers and reference files; “unexpected value” messages are displayed to the participant, prompting them to double-check their response. Physicians are instructed to report the most recent consecutive cases (up to 20 cases depending on the specialty) they had treated during the last 7-day period to discourage selective case submission. After the form submission, additional validations and trend checks are performed; anomalous values are discussed with the submitting participant and corrected as needed.

### Patient selection and study variables

Surveys of all patients with neuroendocrine tumors of small intestine, large intestine, stomach and gut (non-pancreatic; C7A.010, C7A.011, C7A.012, C7A.019, C7A.020, C7A.021, C7A.022, C7A.023, C7A.024, C7A.025, C7A.026, C7A.029, C7A.092, C7A.094, C7A.096) completed between January 1st 2017 and March 31st 2021 were available for four European countries: Germany, France, the United Kingdom (UK), and Spain. Variables analyzed in the study were patients age and sex, stage at diagnosis, site of metastases (liver, peritoneum, lung, bones), Ki-67 (< 2, 2–20, > 20) and ECOG performance status (asymptomatic, symptomatic fully ambulatory, symptomatic in bed less than 50%, symptomatic in bed greater than 50% and bedridden).

### Statistical analysis

Baseline characteristics were compared for subjects in different regions using Chi^2^ test for categorical variables and Wilcoxon test for age. *P* values lower than 0.05 were considered statistically significant. All analyses were performed using SAS 9.4 (SAS Institute, Cary, US).

## Results

### Baseline characteristics of study population

Overall, 1354 patients with neuroendocrine tumors of the gastrointestinal tract (GI-NET) were documented in four different European countries. The baseline characteristics of the study population are displayed in Table 1. Out of the analyzed 1354 tumor patients, 535 were found in the UK (39.5%), 289 in Germany (21.3%), 283 in Spain (20.9%) and 247 in France (18.2%). More patients were male than female (53.8% vs. 46.2%) with no significant differences between the analyzed countries. There was a very small but significant difference in average age in patients diagnosed in Spain (61.4 years) compared to France (65.8 years), Germany (65.6 years) and UK (65.7 years). Overall, most patients were identified in the age groups of 61–70 years (31.0%) and 71–80 years (30.7%). Interestingly, the ECOG performance states significantly varied between the different countries with patients in Spain displaying the most favorable performance status (47.7% ECOG 0 and 52.3% ECOG 1–4), while patients in Germany displayed the least favorable performance status (18.0% ECOG 0 and 82% ECOG 1–4), respectively.

**Table 1:** Baseline characteristics of study patients

Variable	Total	France	Germany	Spain	UK	*p* values
*N*	1354	247	289	283	535	
Age (mean, SD)	64.8 (12.0)	65.8 (13.2)	65.6 (10.5)	61.4 (11.5)	65.7 (12.0)	< 0.001
Age group						
16–20	0.3	1.6	0	0	0	< 0.001
21–40	0.4	0.4	0	0.6	0.7	
31–40	2.4	2.8	1.4	3.2	2.4	
41–50	9.3	7.7	6.6	13.8	9.2	
51–60	17.6	16.2	19.0	28.3	17.6	
61–70	31.0	31.2	44.3	32.9	31.0	
71–80	30.7	30.8	19.0	17.7	30.7	
> 80	8.6	9.3	9.7	3.5	8.6	
Women	46.2	50.6	43.6	50.5	43.4	0.087
Men	53.8	49.4	56.4	49.5	56.5	
Stage at diagnosis						
I	1.1	2.0	1.4	1.1	0.6	0.313
II	5.0	7.3	5.2	6.4	3.0	0.037
III	9.3	13.0	8.3	6.4	9.7	0.064
IV	84.6	77.7	85.1	86.2	86.7	0.010
Site of metastases						
Liver	75.3	73.3	68.9	78.1	78.1	0.015
Peritoneum	20.5	20.7	25.6	19.1	18.5	0.099
Lung	16.6	20.2	22.8	13.8	13.1	< 0.001
Bones	9.0	14.6	4.5	8.1	9.4	< 0.001
Ki-67						
< 2	19.5	14.7	6.3	6.9	35.9	< 0.001
2–20	59.8	66.5	52.7	73.3	52.9	
> 20	20.6	18.8	40.9	19.8	11.2	
ECOG performance status						
0-asymptomatic	30.4	31.6	18.0	47.7	27.3	< 0.001
1-symptomatic fully ambulatory	54.7	53.0	51.9	47.7	60.8	0.002
2-symptomatic in bed less than 50%	13.4	12.6	27.7	4.2	11.0	< 0.001
3-symptomatic in bed greater than 50%	1.4	2.4	2.4	0.4	0.9	0.067
4-bedridden	0.1	0.4	0.0	0.0	0.0	0.214

### Site of tumor origin and tumor differentiation

**Fig. 1:**
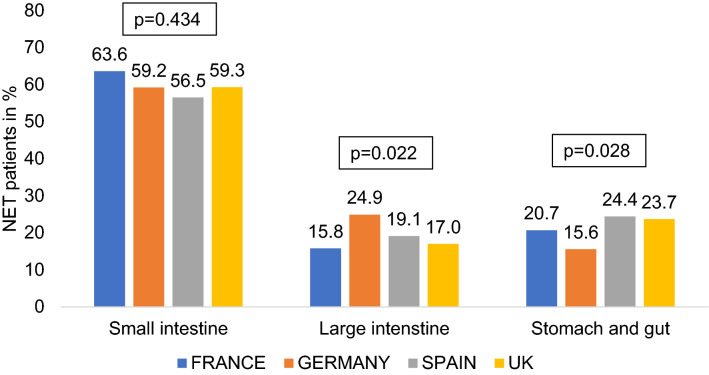
Cancer types of study patients by region

Overall, the majority of patients had a tumor origin within the small intestine (France 63.6%, Germany 59.2%, Spain 56.5%, UK 59.3%; Fig. 1). Interestingly, the proportion of patients with a NET originating from the large intestine was significantly higher in Germany than in all other countries (Germany 24.9%, France 15.8%, Spain 19.1%, UK 17.0%), while patients from Germany displayed significantly lower rates of NET from the stomach (Germany 15.6%, France 20.7%, Spain 24.4%, UK 23.7%). The biologic behavior and prognosis of NET mainly depends on the tumor differentiation. Most patients in our analysis displayed a NET G2 (Ki-67 2–20%). However, there were important differences between the different countries: patients from Germany displayed the lowest rates of well-differentiated tumors (G1, Ki-67 < 2%: 6.3%) but the highest rates of NET G3 (Ki-67 > 20%: 40.9%), while all other countries had more G1 tumors but less G3 tumors (France: 19.5% vs. 20.6%, Spain: 6.9% vs. 19.8%, UK: 35.9% vs. 11.2%).

### Tumor stage and pattern of metastases

We finally compared the tumor stage at the time of diagnosis between the different countries. In the whole population, almost all patients were diagnosed in a metastasized disease stage (84.6%). Notably, in France the number of patients with stage IV disease was lower than in all other countries (77.7%, Germany: 85.1%, Spain: 86.2%, UK: 86.7%). With regard to the site of metastases, the liver represented the most common site of tumor metastases (75.3%), followed by the peritoneum (20.5%), lung (16.6%) and bones (9.0%). Notably, we observed significant differences when the pattern of metastases was compared between the different countries: patients from Germany displayed the lowest rate of liver and bone metastases, while the proportion of patients with lung and peritoneal metastases was higher than in all other countries.

## Discussion

Gastrointestinal (non-pancreatic) neuroendocrine tumors represent a rare but in recent years more frequent tumor entity (Rindi and Wiedenmann 2020). The diagnosis requires the availability of technically advanced procedures, which could potentially distort the measured epidemiology of these tumors (Pavel et al. 2020). Therefore, we compared clinical and histopathological characteristics of patients diagnosed with a GI-NET in four different European countries, all of which feature highly developed healthcare systems. In our analyses including 1354 patients, we found that, although patient characteristics are comparable between countries, there are notable regional differences, e.g., in tumor grading and tumor origin.

One of the major findings of our study is that more than 80% of patients with GI-NET are diagnosed in a metastasized disease stage with the liver representing the most common site of metastases. This finding is highly relevant because for almost all patients with stage IV disease no curative therapy options are available, and the life expectancy of many of these patients is reduced accordingly (Strosberg et al. 2015). For many patients with metastatic NET, the administration of somatostatin analogs represents the current standard of care. In addition to an antisecretory effect, this therapy is also considered to have antiproliferative properties, and in numerous studies the time to tumor progression and overall survival were better than with placebo therapy (Stueven et al. 2019). Newer therapeutic options include the administration of targeted therapies such as the mTOR antagonist Everolimus. In contrast, cytotoxic chemotherapy is ineffective in GI-NET. Just recently, the so-called peptide receptor radionuclide therapy (PRRT) was established as a novel therapeutic option in NET patients refractory to other treatments (Kratochwil et al. 2015; Stueven et al. 2019). Despite this progress, the prognosis in metastatic disease remains poor. Our data, therefore, point to the need for intensive efforts for early detection of neuroendocrine tumors. Such efforts could lead to detection of the disease at earlier disease stages and, thus, greatly simplify and improve the clinical management of these patients.

Another major finding of our study is that a significant proportion of patients with GI-NET feature a G3 histology. Such G3 differentiated neuroendocrine tumors (G3 NET) have to be distinguished from low-to-intermediate grade (G1–G2) NETs, as well as from highly malignant, poorly differentiated neuroendocrine carcinomas (NEC) (Pavel et al. 2020). Today, limited data exist on high-grade neuroendocrine tumors (NETs G3) which represent a new category among neuroendocrine neoplasms (NEN) (Kasajima et al. 2021). Recently, Kasajima et al. analyzed 1513 NEN from a consultation series regarding prevalence, tumor origin, and metastases (Kasajima et al. 2021). Based on the WHO classification of digestive system tumors, 130 NET G3 (9%) were identified, which is similar to the frequency of NET G3 in our analysis. Notably, in our database, 9.3% of all G3 tumors were found in the small intestine, 46% within the large intestine and 26.7% within stomach and gut. Due to the rarity of NET G3, there are currently no data from randomized trials on the optimal treatment of this entity (Pavel et al. 2020). However, there is consensus that cytotoxic chemotherapy should be administered. However, the objective response rate (ORR) with cisplatin-based chemotherapy in NET G3 (Ki-67 < 55%) is much lower than in NEC and cisplatin/etoposide is not recommended and temozolomide-based chemotherapies or STZ-based chemotherapy in the case of pancreatic origin are mostly recommended (Pavel et al. 2020). Nevertheless, the prognosis of patients with G3 NET is much worse compared to patients with well-differentiated NET (G1/G2). Our data regarding regional differences in the frequency of G3 differentiation among NET from different countries might be explained by a different awareness for this specific entity (Pelosi and Travis 2021). Moreover, in Germany, well-differentiated NEN (G1/G2) are mostly treated by endocrinologists, poorly differentiated NEN (G3) rather by oncologists. Due to the design of the database, it seems likely that G3 NET are artificially overrepresented in the German arm of the analysis. Nevertheless, since both biological behavior and optimal treatment is tremendously different from other NET, our results should trigger further efforts to optimize the clinical management of patients with G3 NET.

The main strength of the present study is the use of data from a large number of patients from several countries, enabling the understanding of the intercountry variation. However, our study is subject to some limitations, some of which are specific to NET, others of which reflect general imitations of the database. Regarding NET, data on a potential hereditary background are lacking. Moreover, no data on the functional activity are available. Finally, it is impossible to fully exclude that NEC G3 have been misclassified as NET G3; thus, it seems possible that the database might not be representative for the whole spectrum of GI-NET. Regarding the database, it is important to note that only drug-treated patients were included and that the original questionnaire was not designed for the concrete research purpose. Missing variables such as genetic factors and socioeconomic status are further limitations. Finally, no causal relationships but only associations can be estimated in studies like this and comparison with other established data bases are lacking. Nevertheless, the database was used for many different studies and has demonstrated it suitability for research purpose in many different clinical analyses. Therefore, the differences in clinical and histopathological characteristics identified within this analysis should trigger further epidemiological research allowing to better understand the pathophysiology of NET and to optimize the management of patients with these tumors in different European countries.
